# Supplementary figures and images for: Change of Gut Microbiota in PRRSV-Resistant Pigs and PRRSV-Susceptible Pigs from Tongcheng Pigs and Large White Pigs Crossed Population upon PRRSV Infection
Source: Animals (Basel). 2022 Jun 9;12(12):1504. doi: 10.3390/ani12121504 (PMC9219425; doi:10.3390/ani12121504)

### Rank Abundance Distribution Curves

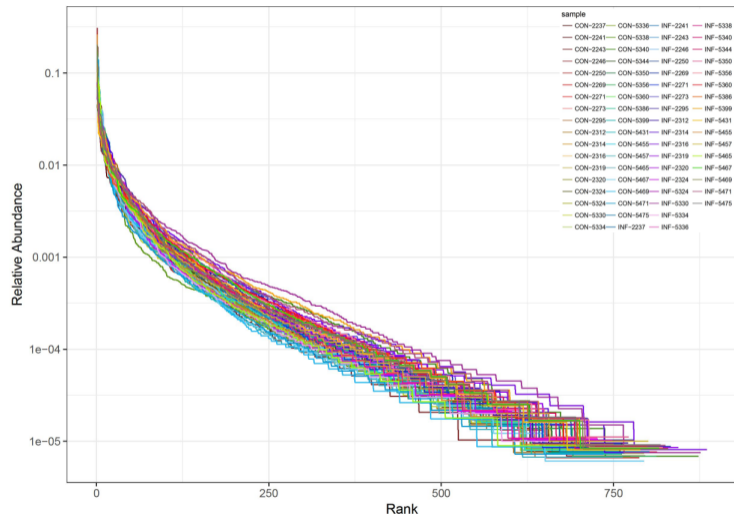

Supplement: Supplementary file 1 [file animals-12-01504-s001.zip › Supplementary figure S1.pdf]

**A**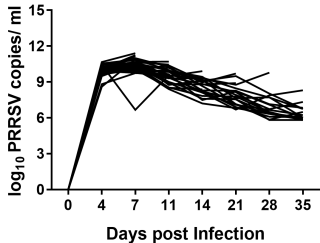**B**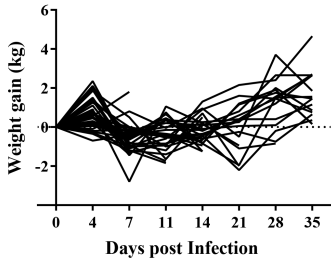

Supplement: Supplementary file 1 [file animals-12-01504-s001.zip › Supplementary figure S2.pdf]
